# Paraphrenia in modern times? Revisiting an elder concept

**DOI:** 10.1192/j.eurpsy.2021.2114

**Published:** 2021-08-13

**Authors:** M. Jesus

**Affiliations:** Centro De Responsabilidade Integrada De Psiquiatria, Centro Hospitalar e Universitário de Coimbra, Coimbra, Portugal

**Keywords:** Paraphrenia, psychosis, elderly schizophrenia

## Abstract

**Introduction:**

The concept of paraphrenia was first introduced by Kraeplin and has since been a controversial issue. However, a group of patients still represent a diagnostic problem and many remind us of the initial description of Paraphrenia: “The uncertain group between paranoia and dementia preacox”.

**Objectives:**

Revisit paraphrenia and to transpose it to modern times.

**Methods:**

Clinical report and literature review.

**Results:**

“M”., a 68 yo women with no psychiatric history was admitted in with depressive humor, anhedonia, asthenia and structured delusional ideas of guilt and persecution and auditory hallucinations. Antidepressant therapy improved the mood, but with worsening of the psychotic symptoms. With further exploration it was was clear that the mood disorder was secondary to the psychotic symptoms that arose in insidiously. The family described her as very reserved and suspicious and notice that she abandoned many of her daily tasks. MMSE was 26 points and the laboratory results and the Cranial Computed Tomography were normal. There was little response to antipsychotics and the patient is undergoing electroconvulsive therapy with positive results.

**Conclusions:**

Initially thought to be a depressive episode, the psychotic symptoms were the primary manifestation. Although the insidious installation, structured delusional ideas and the preservation of the affects pointed to a delusional disorder, the presence of auditory hallucinations and gradual loss of functionality are characteristic of schizophrenia. Some authors rejected the classic definition of Paraphrenia, but accepted that schizophrenia in the elderly could assume a paraphrenic form. In this case, the clinical picture and evolution are close to the classical description of the disorder.

**Disclosure:**

No significant relationships.

